# Di-Tyrosine Cross-Link Decreases the Collisional Cross-Section of Aβ Peptide Dimers and Trimers in the Gas Phase: An Ion Mobility Study

**DOI:** 10.1371/journal.pone.0100200

**Published:** 2014-06-19

**Authors:** Ewa Sitkiewicz, Jacek Olędzki, Jarosław Poznański, Michał Dadlez

**Affiliations:** 1 Institute of Biochemistry and Biophysics, Polish Academy of Science, Warszawa, Poland; 2 Institute of Genetics and Biotechnology, Biology Department, Warsaw University, Warszawa, Poland; University of Florida, United States of America

## Abstract

Oligomeric forms of Aβ peptide are most likely the main synaptotoxic and neurotoxic agent in Alzheimer’s disease. Toxicity of various Aβ oligomeric forms has been confirmed *in vivo* and also *in vitro*. However, i*n vitro* preparations were found to be orders of magnitude less toxic than oligomers obtained from *in vivo* sources. This difference can be explained by the presence of a covalent cross-link, which would stabilize the oligomer. In the present work, we have characterized the structural properties of Aβ dimers and trimers stabilized by di- and tri-tyrosine cross-links. Using ion mobility mass spectrometry we have compared the collisional cross-section of non-cross-linked and cross-linked species. We have found that the presence of cross-links does not generate new unique forms but rather shifts the equilibrium towards more compact oligomer types that can also be detected for non-cross-linked peptide. In consequence, more extended forms, probable precursors of off-pathway oligomeric species, become relatively destabilized in cross-linked oligomers and the pathway of oligomer evolution becomes redirected towards fibrillar structures.

## Introduction

Alzheimer’s disease (AD) poses a serious challenge to social care systems worldwide, thus justifying studies of the mechanisms underlying AD pathology and the nature of the neurotoxic agent. Several lines of evidence [1] point to a causative link between the aggregated forms of Aβ peptide, derived from proteolysis of amyloid precursor protein (APP), and AD, forming the basis for the amyloid cascade hypothesis [2]. The current version of this hypothesis [3] concentrates on a soluble, though non-monomeric, pool of Aβ. The level of cognitive disability appears to have a better correlation with the level of soluble oligomers than with the aggregated amyloid burden [4], [5]. Additionally, oligomers extracted from the tissues of AD patients [6]–[8] exert neuro/synaptotoxicity in a wide range of experimental conditions. The “oligomer hypothesis” is at present central for AD-related research and has been the subject of frequent reviews [9]–[12]. Aβ oligomeric assemblies ranging from dimers to high molecular weight (HMW) [9] have been identified both endogenously and in *in vitro* preparations. It has not been resolved which of those is the major neurotoxic agent. A systematic characterization of biophysical and structural variants of identified oligomeric forms and their correlation with biological effects [9] is therefore a necessity. Studies by many research groups have identified significant differences in the activity of oligomers obtained by incubation of synthetic Aβ and 22 those derived from cell cultures or extracted from the tissues of human AD patients. Synthetic oligomers exert their synaptotoxic/neurotoxic effect at concentration 2–4 orders of magnitude greater than those observed for brain- or cell-derived oligomers [13]–[15]. When measured by their cognitive effects, cell-derived oligomers were also more potent [16]. Gel filtered cell-secreted oligomers are 4000-fold more active than the corresponding synthetic forms in cofilin-actin rod formation, an AD stress-response indicator [17].

A striking feature of the Aβ oligomers originating from cell culture or from the tissues of AD patients is their stability in SDS, which suggests the presence of some stabilizing factor, i.e. a covalent cross-link. This hypothesis has been challenged by the observation of Bitan et al. [18] that SDS itself can artificially induce oligomerization. Other groups have confirmed this conclusion [19], [20], but it was also found that such artificial forms become disaggregated upon boiling in 1% SDS. Also, it is known that species resolved by SDS-PAGE differed markedly in terms of size and stability depending on the source of the oligomer: endogenous or synthetic [19], AD-derived or extracted material from transgenic mouse AD models [21]. Different endogenous Aβ oligomer preparations, when resolved in gels, demonstrated strikingly different distribution of forms [17], [22], despite the fact that they were all similarly treated with SDS. Thus, the SDS influence cannot fully explain the presence of SDS-stable Aβ oligomers in endogenous preparations.

Aside from the SDS controversy, examining the differences in toxicity of *in vitro* and *in vivo* derived oligomers may advance our understanding of the etiology of the disease. The nature of the factor stabilizing the synaptotoxic/neurotoxic forms in the endogenous oligomeric pool remains unknown. Suggested factors include non-covalent stabilization by an unknown protein or lipid, as well as covalent stabilization of dimers and/or higher oligomers by a cross-link. Several types of cross-links which can occur in the case of Aβ oligomers have been suggested. All of these cross-link types may affect toxicity, but it is also possible that one of them plays a crucial role in the stabilization of oligomers. The most frequently discussed cross-links are: a) a covalent bond between the glutamyl side chains and the ε-amino groups of lysine induced by tissue transglutaminase (TGase) [23], [24]; b) a cross-link generated by a major product of unsaturated fatty acid oxidation, 4-hydroxynonenal (4-HNE) [25], [26]; and c) oxidation-mediated di-tyrosine (di-Tyr) formation [27]. K16 and Q15 are juxtaposed in the fibril structure and transglutaminase-catalyzed cross-links have been found to colocalize with Aβ in senile plaques in the brains of AD patients [28]. In neuroblastoma cell culture, TGase activity was found to increase Aβ-induced cell death [29]. Also 4-HNE was elevated in the brain [30], [31] and plasma [32] of Alzheimer’s patients and co-localized with Aβ amyloid deposits in affected tissue [33]. AD-specific lipid oxidation is the effect of Aβ-induced oxidative stress, one of the hallmarks of AD. The source of the oxidative stress is attributed to Aβ-mediated reduction of bound Cu or Fe ions and subsequent production of hydrogen peroxide [34], [35]. As a by-product of these processes, tyrosine radicals are created, which may react with each other, resulting in the formation of di-Tyr cross-linked Aβ [27]. Several fold increases in the levels of di-Tyr cross-links have been found in the hippocampus and neocortex of AD patients [36], directly localized to plaques and fibrils [37].

A single tyrosine occupies position 10 in human Aβ, while in rodents this residue is substituted by phenylalanine. The Phe10 version of Aβ does not form dimers or other SDS-stable oligomers on its own [24]. The Ala10 version of recombinant Aβ was found to be non-toxic and unable to induce oxidative stress responses in cell culture [38]. The reaction of the two Tyr10 radicals may be facilitated in the case of Aβ oligomers by the proximity of the two tyrosine residues, which is the case for oligomers with parallel register of the polypeptide chains. Indeed, the formation of di-Tyr in Aβ was easily induced *in vitro* mediated either by copper ions [37], [39], [40], or by peroxidase [41]. Incubation of neuroblastoma cells with monomeric Aβ1-42 is sufficient to obtain its di-Tyr linked forms efficiently internalized into the cells [37]. Induction of di-Tyr in synthetic Aβ1-42 preparations led to a marked, 620-fold increase in the number of cofilin-saturated actin bundles [17], an alternative histopathological AD hallmark [42] and in Aβ1-40 to increased neurotoxicity in a neuronal cell line viability assay [43].

To obtain insight into the structural properties of oligomers, analytical methods are needed that can distinguish signals from different oligomeric forms, as these frequently coexist in solution and rapidly interconvert. One such method is mass spectrometry combined with ion mobility separation [44], which enables the measurement of the collisional cross section values (Ω) of different oligomeric forms. Previously [45], we have characterized a pool of non-covalently stabilized Aβ1-40 oligomers in terms of their Ω values. In the present work, we have prepared a di-Tyr-stabilized pool of oligomers and measured the impact of di-Tyr link on the Ω value of dimeric and trimeric forms of Aβ1-40. We demonstrate that the presence of the di-Tyr cross-link stabilizes compact dimeric and trimeric forms thus leading to the decrease of the population of more extended forms.

## Results

### Crosslinking of Aβ1-40 by a di-Tyrosine Bond

**Figure 1:**
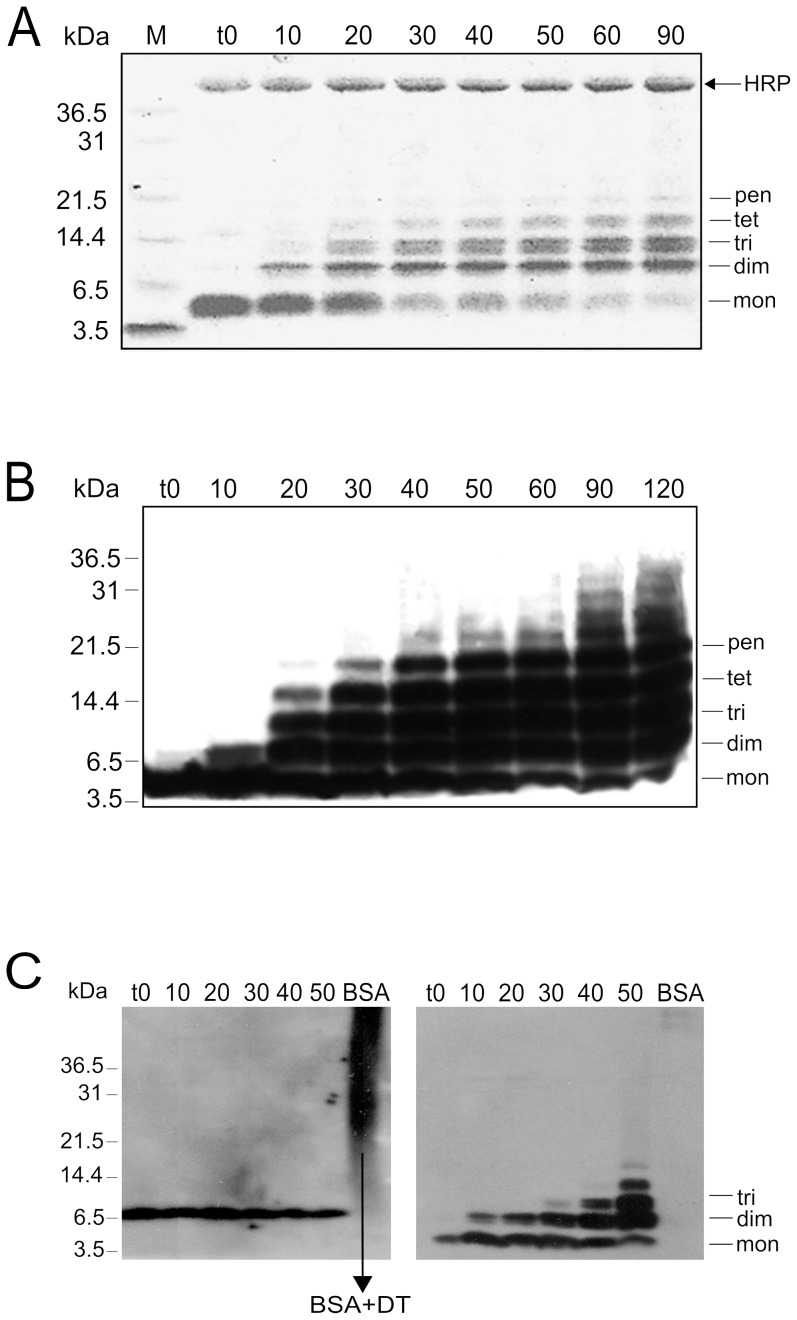
SDS PAGE of Aβ oligomers. SDS PAGE of Aβ oligomeric forms obtained at different times of incubation with H_2_O_2_ and horseradish peroxidase and visualized by (**A**) Coomassie staining, (**B**) anti-Aβ antibodies. (**C**) Gel stained with anti-di-Tyr antibody (left panel) at different reaction times shows the expected signal corresponding to the mass of a dimer. A gel developed with an anti-Aβ monoclonal antibody 6E10 (right panel). Arrow indicates the position of the BSA containing Di-Tyr cross-link (as a control). See also **Figure S1**.

In the presence of H_2_O_2_ and horseradish peroxidase (HRP), SDS-stable oligomeric species of Aβ1-40 formed readily. The reaction was followed by SDS PAGE of aliquots collected at different times points (**Figure 1**). At 20 min after H_2_O_2_ addition, even in a relatively low (20 µM) concentration of hydrogen peroxide, a single, monomeric gel band splits first into the dimeric, and subsequently to the trimeric, tetrameric and pentameric forms. Both Coomassie staining (**Figure 1A**) and Western blots with anti-Aβ antibodies (**Figure 1B**) showed similar patterns. However, on a Western blot, an additional weak band corresponding to hexamers and higher oligomers could also be detected. Similar distribution of oligomers during di-Tyr formation was observed before for Aβ1-42 [37]. When HRP was omitted from the reaction, no oligomeric band could be detected (**Figure S1A**) indicating that the observed SDS-stable oligomeric species were not artificially induced by SDS. When H_2_O_2_ was not added to the reaction, a weak dimeric band could be detected at longer reaction times (**Figure S1B**).

**Figure 2:**
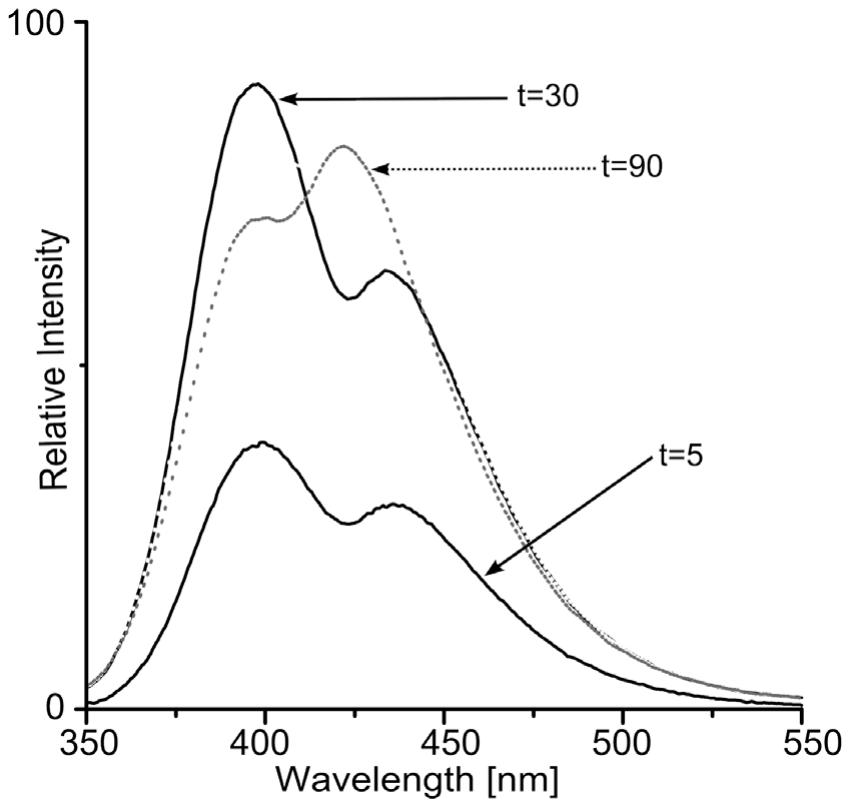
Di-tyrosine fluorescence spectra. Fluorescence spectra collected at different times of incubation of Aβ with H_2_O_2_ and horseradish peroxidase, upon excitation at 315 nm. The two bands at 400 nm and 425 nm correspond to di-Tyr and tri/tetra-Tyr, respectively. Arrows indicate time of reaction in minutes.

Earlier studies describe the formation of di-Tyr covalent bonds in Aβ1-40 peptide under conditions used in our experiment [46]. Nevertheless, to confirm the identity of the cross-link we have carried out two control experiments. Western blots with anti-di-Tyr antibodies 1C6 directed against di-tyrosine residues (**Figure 1C**) confirmed the formation of the di-Tyr cross-link in Aβ1-40 dimers. Also, a characteristic fluorescence spectrum (λ_ex_ = 315, λ_em_ = 400 nm) accompanied the appearance of SDS-stable oligomeric bands on SDS-PAGE gels (**Figure 2**), marking the presence of di-Tyr bond. Time-dependent changes in the fluorescence were observed. At 30 min of the reaction, the amplitude of the fluorescence ca. 400 nm reached its maximum value and after that it decreased. Simultaneously, a shoulder with emission maximum ca. 425 nm began to rise in the spectrum. The band at 425 nm was expected for tri-Tyr and/or tetra-Tyr cross-links [47], so presumably at later times of the reaction, di-Tyr cross-links were converted to tri- or even tetra-Tyr. SDS gel band densitometry showed a steady increase in the intensity of oligomeric bands over the course of the reaction, that is why the decay of the fluorescence signal at 400 nm could not be caused by the decrease of the quantity of oligomeric forms.

**Figure 3:**
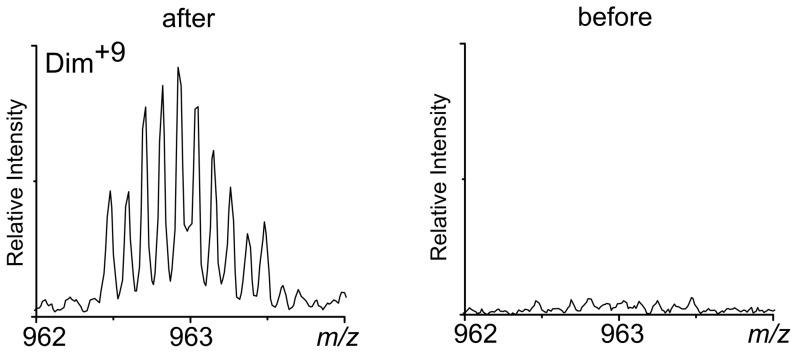
Spectra for covalently stabilized DIM^9+^. Fragments of mass spectra collected before (right panel) and after 90 min of reaction (left panel). Shown is the fragment of spectra in which a new signal, absent before reaction, was observed. This signal corresponded to 9+ charged dimer at 963 m/z.

In agreement with the fluorescence measurement results, mass spectrometry (MS) verified the presence of not only di-Tyr cross-links, but also tri-Tyr cross-links. Also, the measured mass shifts indicated that the expected tyrosine cross-links (the loss of two hydrogen atoms per di-Tyr bond) were the only modifications present in the peptide. The MS spectrum collected before the start of the reaction contained signal from the monomer, as well as oligomeric signals from species stabilized only by non-covalent interactions, not stable in the SDS, with molecular masses equal to multiples of the monomeric mass. In the course of the H_2_O_2_/HRP reaction, a small but measurable decrease in the molecular mass of oligomers was observed and also a unique signal corresponding to a dimeric form bearing charge 9+ appeared (**Figure 3**). The mass decrease calculated from the spectra for each form (**Table 1**) indicated a loss of 2 Da per each monomer cross-linked to oligomer. In other words, for the di-Tyr cross-linked dimer the mass is smaller by 2 Da than that calculated for a non-covalent oligomer form, containing two intact Aβ1-40 molecules. For the trimer, the difference is 4 Da. MS measurements confirmed the coexistence of both di-Tyr and tri-Tyr forms, responsible for the presence of higher-order SDS-stable oligomeric forms.

**Table 1:** Experimental values of molecular masses (M_B_, M_A_), IMS drift times (t_D_) and collisional cross-section (Ω) for dimer and trimer.

1	2	3	4	5	6	7	8	9	10
	BEFORE REACTION WITH HRP AND H_2_O_2_				AFTER REACTION WITH HRP AND H_2_O_2_				
z	Av. m/z	M_B_	t_D_	Ω	Av. m/z	M_A_	t_D_	Ω	M_A_ – M_B_
**DIMER**									
3	2887.05	8658.15	17.20	1018.1	2886.88	8657.64	17.20	1018.1	−**0.51**
4	2165.82	8659.28	10.36	975.4	2165.69	8658.76	10.36	975.4	−**0.52**
5	1732.90	8659.50	10.70	1245.1	1732.91	8659.55	10.70	1245.1	0.05
5	1732.91	8659.55	10.15	1203	1732.92	8659.60	10.15	1203	0.05
5	1732.90	8659.50	7.39	979	1732.62	8658.10	7.39	979	−**1.4**
6	1444.28	8659.68	9.37	1370	1444.28	8659.68	9.37	1370	0
6		8659.68			1443.93	8657.58	7.50	1185	−**2.1**
7	1238.09	8659.63	8.16	1460	1238.10	8659.70	8.16	1460	0.07
7	1238.07	8659.49	7.60	1394.7	1237.82	8657.74	6.95	1315	−**1.89**
8	1083.47	8659.76	6.28	1407.5	1083.46	8659,68	6.28	1407.5	−0,08
8		8659.76			1083.26	8658.08	7.17	1534.5	−**1.68**
9		8659.76			962.97	8657.73	6.06	1547	−**2.03**
**TRIMER**									
4	3247.38	12985.52	16.60	1326	3247.19	12984.76	16.60	1326	−**0.76**
5	2598.63	12988.15	11.25	1286.5	2598.44	12987.20	11.25	1286.5	−**0.95**
6	2165.79	12988.75	12.30	1636.3	2165.69	12988.14	12.30	1636.3	−0.60
6	2165.82	12988.92	8.60	1295.8	2165.60	12987.60	8.60	1295.8	−**1.32**
7	1856.60	12989.20	12.20	1898.9	1856.59	12989.13	12.0	1878.5	−0.07
7	1856.58	12989.06	10.25	1695	1856.41	12988.15	10.14	1683	−**1.19**
7		12989.06			1856.07	12985.45	8.36	1484	−**3.57**
7		12989.06			1856.04	12985.28	7.60	1394.7	−**3.78**
8	1624.67	12989.36	11.03	2032.1	1624.68	12989.44	11.03	2032.1	0.08
8	1624.68	12989.44	10.25	1937	1624.45	12987.60	9.70	1868.7	−**1.84**
8		12989.44			1624.43	12987.44	9.04	1784.8	−**2.00**
8		12989.44			1624.18	12985.44	7.64	1599.4	−**4.00**
10	1299.92	12989.20	9.04	2231	1299.77	12987.70	8.05	2068.6	−**1.50**
10		12989.20			1299.53	12985.30	7.17	1918	−**3.90**

Molecular masses (M_B_, M_A_), drift times (t_D_) and collisional cross-section (Ω) values corresponding to dimeric (upper table) and trimeric (lower table) forms detected before (columns 2–5) and after (columns 6–10) reaction with HRP and H_2_O_2_. Species are denoted by their charge state z (column 1) assigned by the analysis of the isotopic envelope peak spacing. This allowed a direct calculation of the molecular mass of the species (column 3 and 7) from the average m/z (columns 2 and 6). HRP/H_2_O_2_ reaction results in di-Tyr and tri-Tyr covalent cross-link formation with concomitant molecular mass loss of 2 Da per each bond formed. This is illustrated by the observed loss of the mass of the oligomeric form after reaction (column 10). The presented results are mean values from two independent experiments. The maximum difference for m/z (columns 2 and 6) was ±0.025, corresponding to the largest difference of mass values of ±0.175 Da (columns 3 and 7). The largest difference in drift time values was 0.11 for dim^8+^ signal (dt 6.28) which corresponds to the difference of CCS values of 1.1%. In all other cases the discrepancy between two replicate measurements was lower than 0.9%.

### IMS-MS Analysis of Cross-linked Aβ1-40 Oligomers

Mass spectrometry, when coupled with ion mobility separation (IMS), allows the resolution of signals not only in the domain of the molecular mass, but also in the domain of the collisional cross-section (Ω), resulting in a two dimensional spectrum. During ion mobility separation, the ions drift in the gas-filled analyzer and the time of drift is proportional to their collisional cross-section. Collisional cross-section characterizes the structural properties of the molecule, being smaller if the structure of a given molecule is compact and larger if it is extended.

**Figure 4:**
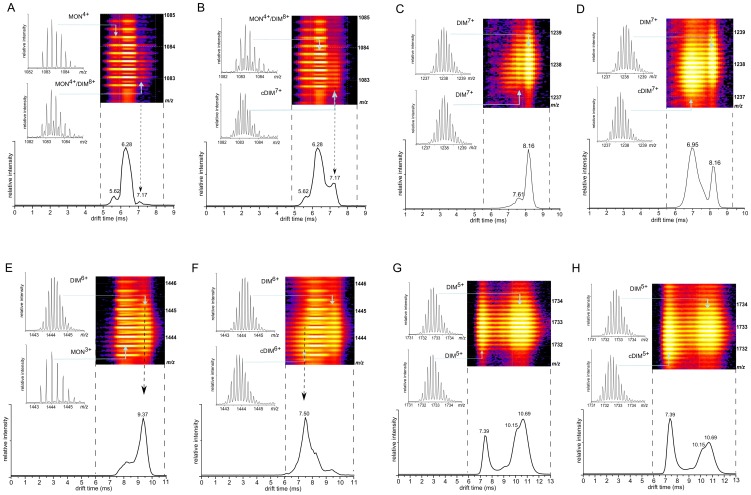
Fragments of IMS-MS spectra for different charged dimer. Selected regions of ion mobility separated mass spectra (IMS-MS) covering dimeric signals in 2D rendition (colored panels, drift time at horizontal axis and m/z at vertical axis). Lower panels show the corresponding ion mobility drift time profiles, i.e., either projections of the signal group on the drift time axis or cross-sections at a different m/z value, as described below. Panels showing isotopic envelopes are cross-sections at indicated (blue arrows) drift time values correspond to a given oligomeric form, for instance DIM^8+^ denoting a non-covalently stabilized dimer charged 8+, whereas cDIM^8+^ is a covalently stabilized dimer charged 8+, etc. Spectra are shown before reaction with H_2_O_2_/HRP (**A,C,E,G**) and at 90 min of reaction **(B,D,F,H**). (**A,B**) 1082–1085 m/z region with overlapping MON^4+^ and DIM^8+^ signals present. The two drift time profiles are collected at m/z values indicated by black arrows, showing a new isotopic envelope after reaction (**B**), corresponding to cDIM^8+^, characterized by a smaller m/z and drift time (7.17 ms) longer than DIM^8+^/MON^4+^ envelope at 6.28 ms. (**C,D**) 1236–1240 m/z region, showing signals of the two structurally alternate (at 7.61 and 8.16 ms) DIM^7+^ forms before reaction (**C**), after incubation (**D**) accompanied by a strong new signal of cDIM^7+^ at smaller m/z and shorter drift time (6.95 ms). (E,F) 1442–1446 m/z region with MON^3+^ and DIM^6+^ signals present. The two drift time profiles are collected at m/z values indicated by black arrows. (**G,H**) 1731–1735 m/z region before reaction (**G**) showing signals of at least four structurally alternate DIM^5+^ forms, with no new forms after incubation (**H**) but with equilibrium shifted towards the most compact of the four forms present before reaction. Experiment was repeated at least three times. See also **Figure S2.**

The IMS-MS spectrum of Aβ1-40 was analyzed in previous work (see Figure 1 in [45]) where it was shown that the spectrum contained an abundance of signals that could be attributed to different monomeric and non-covalently stabilized oligomeric forms. An important feature of these spectra was the coexistence of compact and extended forms of many oligomeric species. In the present work the IMS-MS spectra of unreacted Aβ1-40 were used as a control always preceding the measurements of the products of H_2_O_2_/HRP reaction with the peptide. Upon the reaction with H_2_O_2_/HRP, signals representing covalently stabilized forms appeared that were shifted in the domain of molecular mass and in some cases also in the domain of the drift times, indicating changes of collisional cross section. However, the main changes observed in the drift time profiles were shifts in the relative population of the structurally different forms (compact *vs*. extended) of a given oligomer. In majority of cases these two forms were present also in control spectra, although in some cases the compact form existed as a minor species. New species of unique drift time, absent in control spectra were less frequent. For instance, for one of the charged forms of the dimer (DIM^9+^), the corresponding signal could not be detected in the control spectrum before reaction, but was prominent after formation of the di-Tyr link. Before reaction, the DIM^8+^ form of the dimer co-migrated in the drift chamber with MON^4+^ monomer at 6.3 ms (**Figure 4A**). After reaction, a new isotopic envelope was detected that corresponded to an alternative structural form of the 8+ charged dimer (**Figure 4B**), characterized by a longer drift time of 7.17 ms. Comparison of the drift time profiles and isotopic envelopes (insets in Fig. 4A,B) for MON^4+^ and DIM^8+^ (same m/z of 1083.15) before and after the reaction showed that the reaction product is solely represented by the drift time of 7.17 ms. This result indicated that the di-Tyr-linked form of DIM^8+^ populates only a more extended form than the corresponding non-covalently stabilized dimer. No other changes were observed in drift time profiles for these forms. Due to the partial overlap of the corresponding isotopic envelopes, the mass shift observed upon formation of a di-Tyr bond could be in this case precisely measured. Nevertheless the mass decrease of 1.68 Da in the cross-linked dimer corresponds well with the value of 2 Da, expected in the case of formation of a single di-Tyr bond between the two peptide molecules. In the case of other charge states of the dimer namely 7+ and 6+ the signals of cross-linked species were better resolved and the measured mass decrease was closer to expected. In the case of DIM^7+^ at 1237–1239 m/z (**Figure 4C,D**), the initial distribution of drift times culminating at 8.16 ms, after reaction was accompanied by a new isotopic envelope at 6.95 ms. In this case, the two isotopic envelopes were reasonably well separated and the mass difference between the DIM^7+^ species before reaction and the new species after reaction were measured to be 1.89 Da. Thus, the isotopic envelope at 6.95 ms corresponded to the product of the reaction, a 7+ charged form of Aβ dimer linked by a di-Tyr covalent bond. Unlike in the case of DIM^8+^ signal, this form is characterized exclusively by a shorter drift time, indicating a more compact structure. No mass shift was observed for the isotopic envelope at 8.16 ms after reaction.

A shorter drift time was also observed for di-Tyr linked dimer forms bearing charges 6+ and 5+ (**Figure 4E–H**). For the DIM^6+^ signal, the analysis of the drift time profile after reaction revealed a new isotopic envelope corresponding to a covalent dimeric form, characterized by the drift time of 7.5 ms. Identity of this new species was confirmed by the 2 Da decrease in mass, marking a di-Tyr linked dimer. Interestingly, in the case of DIM^5+^ (**Figure 4G,H**), a di-Tyr linked form drifted with the same time (7.39 ms) as one of the two major non-covalently stabilized forms of the 5+ charged dimer (denoted as “compact” in Figure S3 [45]) strong already in the control experiment before the reaction. In the case of DIM^5+^, formation of a di-Tyr bond did not lead to a new structural form, but rather shifted the equilibrium between two major forms towards a more compact one. Consequently, due to partial overlap of the isotopic envelopes for non-covalent and covalent DIM^+5^, the mass shift (1.4 Da) bond was smaller than expected.

**Figure 5:**
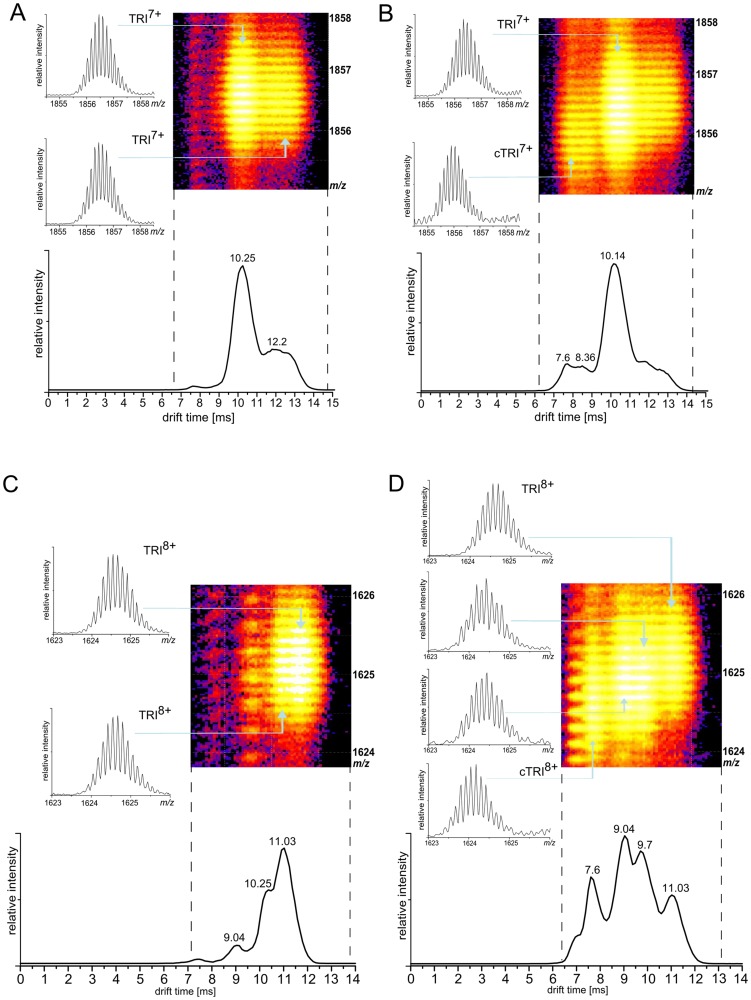
Fragments of IMS-MS spectra for different charged trimer. Selected regions of ion mobility separated mass spectra (IMS-MS) covering trimeric signals in 2D rendition (colored panels, drift time at horizontal axis and m/z at vertical axis). Lower panels show the corresponding ion mobility drift time profiles, i.e. projections of the signal group on the drift time axis. Panels showing isotopic envelopes are cross-sections at indicated (blue arrows) drift time values which correspond to a given oligomeric form, for instance TRI^8+^ denoting a non-covalently stabilized trimer charged 8+, whereas cTRI^8+^ a covalently stabilized trimer charged 8+, etc. Spectra are shown before reaction (**A,C**) and at 90 min of reaction (**B,D**). (**A,B**) 1854–1858 m/z region showing signals of the two structurally alternate (at 10.25 and 12.2 ms) TRI^7+^ forms before reaction (**A**), after incubation (B) accompanied by a strong new signals of cTRI^7+^ at smaller m/z and shorter drift time (8.36 and 7.6 ms), i.e., corresponding to covalently stabilized more compact trimeric form. (**C,D**) 1624–1626 m/z region with two structurally alternate non-covalently stabilized forms of 8+ charged trimer (at 10.25 and 11.03 ms) before reaction, dominated after incubation by new trimeric forms covalently stabilized by a di-Tyr (at 9.04 and 9.7 ms) and a tri-Tyr (at 7.6 ms) bond. Experiment was repeated at least three times. See also **Figure S2.**

For the trimeric forms bearing charges of 4+, 5+, and 6+ a similar mixture of non-covalent and covalent forms with the same drift time was observed. In contrast, for the trimer charged 7+ after reaction a new trimeric isotopic envelope appeared at 7.6 ms (**Figure 5A,B**). This new species was characterized by a mass decrease of 3.78 Da, close to 4 Da expected for a trimer linked by a tri-Tyr bond. Its drift time was shorter than for the corresponding non-covalent TRI^7+^ form observed before reaction. Also, in the case of the TRI^8+^ form, multiple new species with decreased mass and shorter drift time appeared after reaction (**Figure 5C,D**). The form with the shortest drift time of 7.6 ms is characterized by a mass decrease of 4 Da, indicating a tri-Tyr linked trimer. The other two forms, drifting at 9.0 and 9.7 ms, represent two structurally different variants of a trimer with a single di-Tyr link, as their mass decreases were 2 Da and 1.84 Da, respectively. Such trimers must thus consist of one di-Tyr linked dimer accompanied by one non-covalently associated monomer. The 9+ charged trimer signal was obscured by co-migrating stronger DIM^6+^ and MON^3+^ signals, therefore the mass change could not be assessed. For higher 10+ charge, the trimeric signal at 1299 m/z and 7.17 ms appeared, which was barely detectable in the control spectrum before reaction. The other form drifting at 8.05 represented a trimer with one di-Tyr link.

In trimers, similarly to dimers, more compact forms became preferentially populated. However, at highest charges the effect is opposite, i.e. new, extended forms appear which were barely detectable in the control spectra. Although some species appear on the spectrum only after the reaction, the main effect of the cross-linking is shifting the quantitative equilibria within a limited set of structural forms. The set of drift times of oligomeric forms present on the spectrum before the reaction remained largely unaltered after crosslinking. As shown by the Gaussian fitting procedure (**Figure S2**) the effect of the cross-link can be accommodated in the fit only by adjusting the signal amplitudes, reflecting the increase or decline in the abundance of the compact or extended oligomer forms.

**Figure 6:**
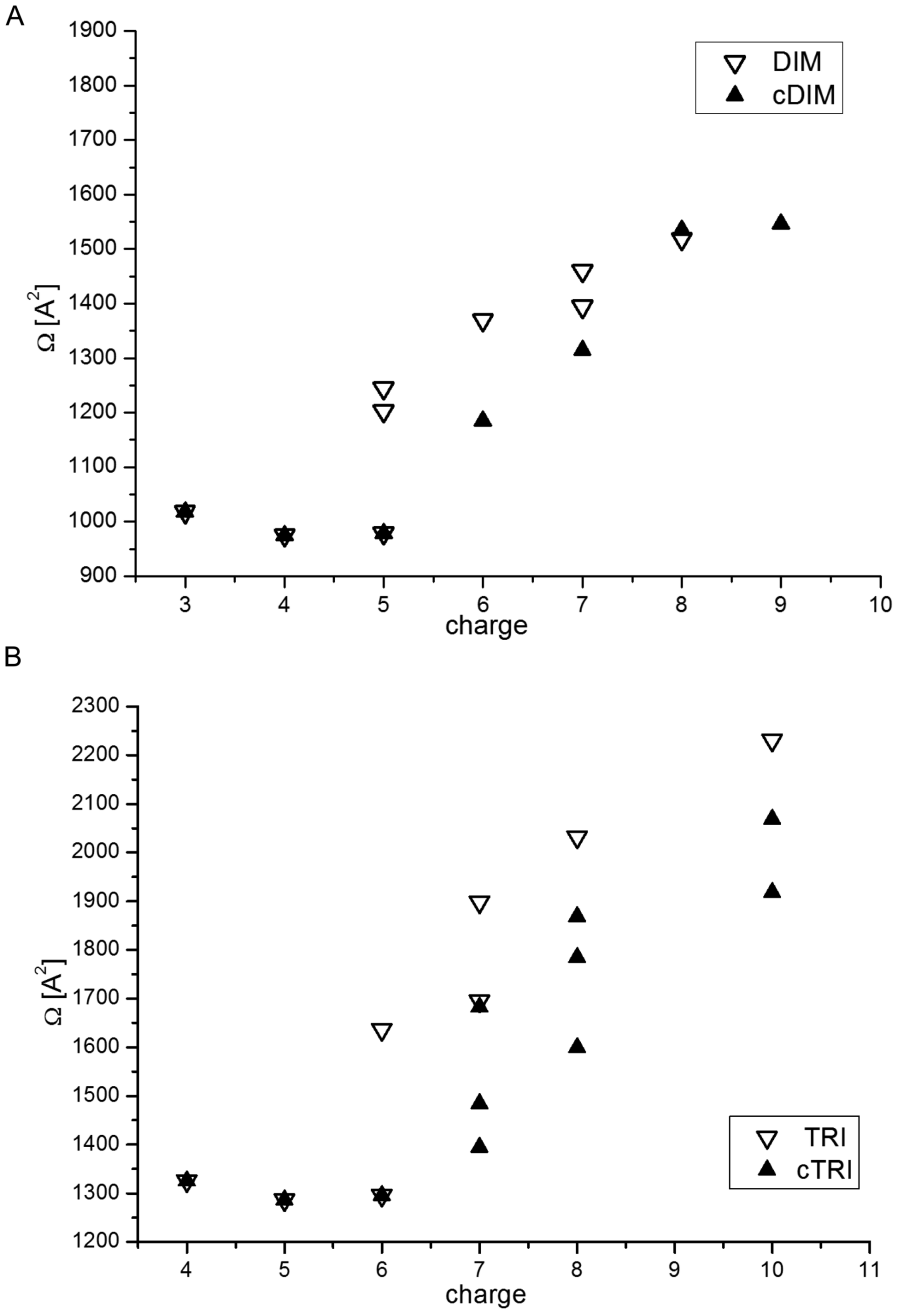
Collisional cross section (Ω) for dimer and trimer. Dependence of the collisional cross-section (Ω) of major dimeric (A) and trimeric (B) forms of Aβ obtained from the analysis of drift time profiles in IMS-MS spectra on the charge of the molecule before reaction (open triangles) and after di−/tri-Tyr crosslinking (solid triangles).

Drift times measured by IMS can be converted to collisional cross-section (Ω) values. The spectra need to be calibrated with the species of known Ω values, to calculate the empirical parameters in the following equation:
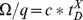
linking the measured drift time (t_D_) value and collisional cross section (Ω). In this equation, q represents the charge of the molecule, c is a parameter which remains unchanged in a single experiment and *X* is the empirically estimated exponent. The two unknown parameters (c, *X*) have to be adjusted for a given experimental setup using a set of molecules of known Ω, and subsequently these values can be used to calculate unknown Ω values. In the present work a standard set of proteins (myoglobin, cytochrome c, ubiquitin) was used as calibrants. Following the protocol from previous work [45] in the present experimental setup we have obtained the best fit between measured and theoretical Ω values for calibrants using values of c = 53.1 and *X* = 0.652. This ensures a reliable interpolation for all t_D_ values in the range from 6 to 17 ms of the drift time. The *X*-value obtained here corresponds well to *X* obtained in previous work [45] and ref. therein. Using the obtained parameters, the Ω values were calculated for all dimeric and trimeric species detected after reaction (**Table 1** and **Figure 6**).

An examination of Ω values for all dimeric forms detected after reaction for charge states from 3+ to 9+ (**Figure 6A**) showed the expected expansion of the molecule with increasing charge caused by strong electrostatic repulsion in the gas phase. In control spectra dimeric forms bearing charges 3+ and 4+ were characterized by single species of similar Ω values of 1018 Å^2^ and 975.4 Å^2^, respectively. In the case of the 5+ charged dimer (**Figure 4G,H**), even in the control sample before reaction, the signal was split into three isotopic envelopes. Two, represented by a stronger group of overlapping drift time signals at 10.15 ms and 10.69 ms, corresponded to more extended states, with Ω value close to 1224 Å^2^, and a weaker one, at 7.39 ms, corresponded to a more compact form with Ω value of 979 Å^2^, similar to 3+ and 4+ dimers. This Ω value was previously [45] assigned as characteristic for the most compact form of the Aβ1-40 dimer. After the reaction, the di-Tyr-linked dimeric species charged 3+, 4+ retain their compact character showing no change of the drift time. The cross-link does not change the structure of the low-charge compact form of the dimer. In contrast, for 5+ charged dimer the cross-linked species populate only the compact form at 7.39 ms and not the extended forms, as no mass shift was observed for the isotopic envelope corresponding to extended forms at 10.15 and 10.69 ms. Similarly, for charges 6+ and 7+, the formation of di-Tyr bond generated compact states (at 7.5 and 6.95 ms, respectively) than the more extended ones observed in control samples (at 9.37 and 8.16 ms, respectively) for which no decrease in mass was observed. Analysis of charge states 3+ to 7+ shows clearly that for cross-linked species only compact forms are populated. It can be concluded that the introduced cross-link stabilizes the compact form of the dimer since it becomes less susceptible to charge-dependent expansion. Interestingly, for the even higher charge of 8+ (**Figure 4A,B**), the di-Tyr link induced the formation of a more extended structure with higher Ω value (represented by signal at 7.17 ms) than the one detected in control spectra (at 6.28 ms). Also, a 9+ charged dimer, absent in the control, was observed only as a di-Tyr crosslink and was characterized by a high Ω value. This effect can be rationalized by assuming that 1460 Å^2^ is a maximum Ω value possible for the non-covalently stabilized dimer, preceding its decomposition to monomers caused by increased charge. So, in the case of the control spectra, the species with higher Ω values were not observed because they were no longer stable due to electrostatic repulsion leading to decomposition of the dimer. With the di-Tyr link present, the decomposition was prevented, the covalent link being responsible for the integrity of the dimer in spite of its high charge. The most extended state of the cross-linked dimer was characterized by the Ω values of ca. 1550 Å^2^.

For trimers (**Figure 6B**), the most compact state with Ω of ca. 1295 Å^2^ was populated at lowest charge states of 4+ to 6+, with common drift times in control spectra and in overlapping signals from mixtures of cross-linked and non-covalently stabilized oligomers after reaction. Similarly to dimers, low charge cross-linked trimers populate compact structural states of unchanged Ω. For trimer 6+ the spectrum is overcrowded due to the presence of DIM^4+^ and MON^2+^ signals, nevertheless the cross-linked trimer could be detected, although it overlapped with non-cross-linked trimer. At higher charges of 7+ to 10+ different forms of shifted Ω, corresponding to di-Tyr and tri-Tyr linked trimers, were observed. Similarly to dimers, both di-Tyr and tri-Tyr cross-linked trimers at intermediate charges populate states of increased compactness, however tri-Tyr cross-link leads to more compact trimers than di-Tyr.

In general, for lowest charges the cross-linked species populate equally compact states to the respective oligomers detected before reaction. The structure of compact dimers and trimers remains unchanged in the presence of a cross-link, but it becomes more stable since for higher charges the shift in equilibrium towards compact states was observed for many forms of cross-linked oligomers. At highest charges new, highly extended cross-linked species appear after reaction. These forms were not observed in control spectra due to inability of non-covalently stabilized oligomers to retain integrity upon stronger charging.

### Molecular Modeling

**Figure 7:**
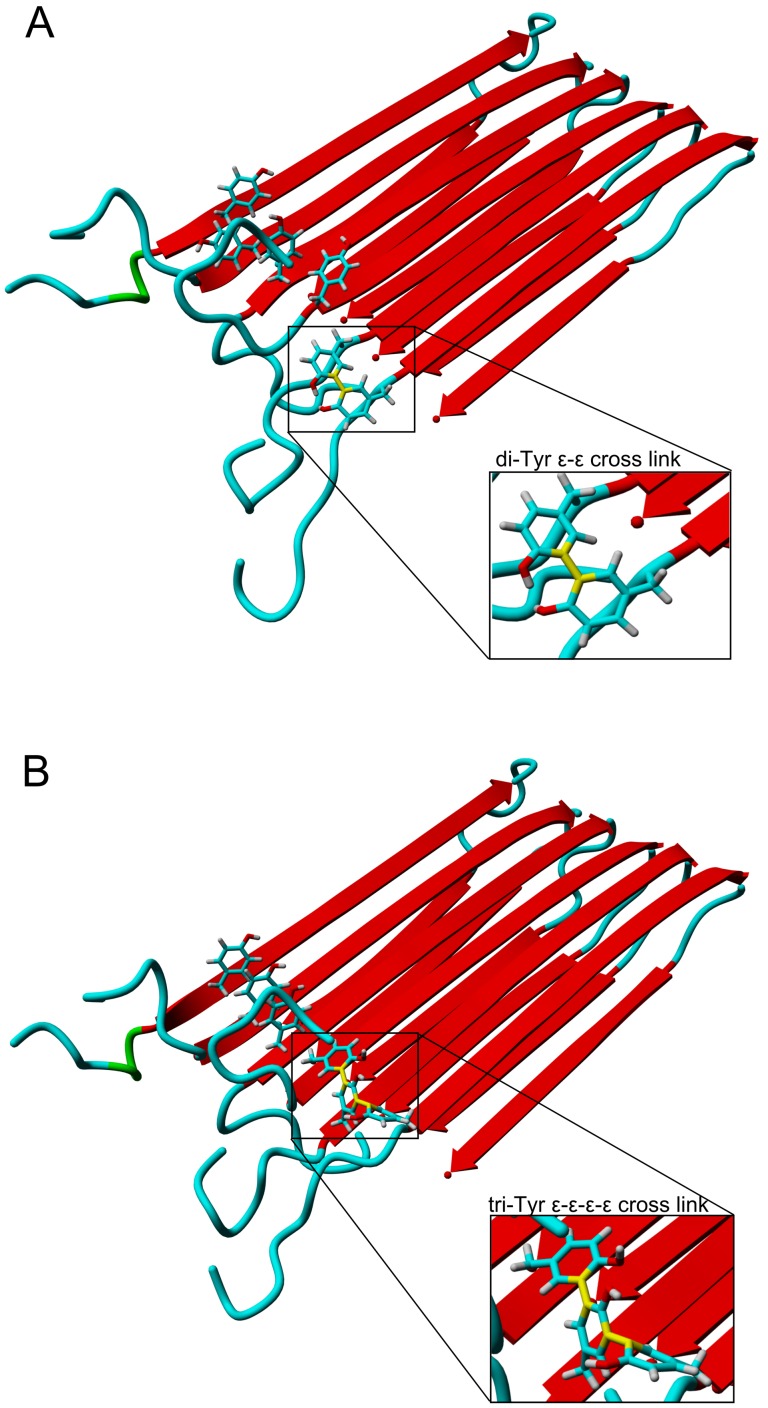
Molecular models of cross-link Aβ oligomer. Molecular models of the compact form of Aβ hexamer cross-linked by di- (A) or tri-Tyr (B) bond.

To verify the compatibility of a cross-link with the molecular model of an oligomer constructed in previous work [45] we have incorporated di-Tyr and tri-Tyr cross-links into the model and tested for integrity using molecular dynamics. For this aim the two or three neighboring Tyr10 residues were converted to di- or tri-Tyr residues, respectively, and new structures were subjected to molecular dynamics. The results of the calculations led to the structures shown in **Figure 7**. Three types of di-tyrosine modifications (i.e., δ-δ, δ-ε, and ε-ε cross-links) were at first introduced between neighboring monomers located at the edge of the hexamer, and the resulting oligomers were subjected for 20 ns MD simulations. All three di-Tyr modifications were consistent with the Aβ oligomer structure, however further simulations showed that ε-ε cross-links induced the smallest distortions (**Figure 7A**). Moreover, only ε-ε-ε-ε tri-Tyr modification (**Figure 7B**) was compatible with the canonical fibril structure, whereas all tetra-Tyr modifications, including ε-ε-ε-ε-ε induced substantial deformation along the axis of oligomer elongation.

The IMS analysis shows that di- and tri-meric Aβ species stabilized by di-Tyr and tri-Tyr bonds tend to populate more compact states than their non-covalently stabilized counterparts. At higher charges new, highly extended forms appear for cross-linked oligomers. These highly extended structures are characteristic for the gas phase since in solution the electrostatic repulsion is decreased by surrounding water dipoles. Observed stabilization of the compact structural forms by a cross-link redirects the evolution of oligomeric structures either along the on-pathway or off-pathway axis, as discussed below, and thus may be of importance for the efficiency of the generation of neurotoxic Aβ species.

## Discussion

Using Ion Mobility-Mass Spectrometry analysis, we investigated the impact of a di-Tyrosine (and tri-Tyrosine) cross-link on the collisional cross-section of oligomers of the Aβ1-40 peptide. In our previous work [45], we discovered the coexistence of compact and extended structural forms of the non-covalent oligomers of the same order. For majority of oligomeric forms introduction of the di-Tyr cross-link leads to increased fraction of compact species that could also be detected in control spectra of non-cross-linked oligomers. In other cases new, more compact forms appear in the spectra after the di-Tyr formation reaction. Only for the highest charge states, the di-Tyr cross-linked oligomers populate forms with highly extended structure. These extended forms are absent in the control spectra of non-covalently stabilized oligomers. With increasing charge, non-covalently stabilized oligomers may undergo decomposition into monomers (an outcome that is not possible in the presence of a covalent cross-link). On the other hand at low charges, non-covalent dimer (e.g. dimer^4+^) does not need to be stabilized by the di-tyrosine and dominates also in the control spectrum.

To our knowledge, presented work is the first attempt to characterize cross-linked Aβ1-40 oligomers in structural terms. In an earlier investigation [39], no structural consequences after di-Tyr formation were observed, but the NMR-based structural study was limited to the very short Aβ fragment 8–14, with any stabilizing effect possibly being too small to be detected. A covalent cross-link in general is expected to stabilize structure in oligomers due to entropic reasons. It has been shown before that Aβ1-40 has an intrinsic tendency to oligomerize in solution, even without covalent stabilization. The presence of non-covalent oligomers might influence the rates of formation of the covalent di-Tyr bond, by decreasing the entropy loss accompanying the formation of a di-Tyr bond in the pre-existing non-covalently stabilized oligomer. Moreover, if the pre-existing oligomer accelerates the di-Tyr formation, the presence of the di-Tyr bond is expected to stabilize the oligomer structure by the same mechanism as the disulfide bonds stabilize the structure of proteins. High efficiency of di-Tyr formation in fibrils was interpreted before in favor of a close juxtaposition of Tyr residues in fibril structures [39].

The stabilizing effect may be exerted independently of the nature of a cross-link. Any cross-link may in principle augment Aβ oligomer’s toxicity by increasing their stability or quantity or facilitating further oligomerization. Following such a line of reasoning, artificially introduced cross-links in the form of disulfide bonds have also been studied. Strong acceleration of the transition to the protofibril form was observed for disulfide-stabilized Aβ oligomers [48]. More importantly, disulfide-stabilized (Aβ(1-40)S26C) oligomers/protofibrils showed markedly increased synaptotoxicity in mouse hippocampus [6], [49]. Covalently linked dimers may exert enhanced toxicity by themselves or may increase stability of higher order forms which incorporate them. Observed here di-Tyr stabilized trimer exemplifies oligomeric forms in which only some of monomers are covalently linked by a cross-link. Such “mixed” trimer, containing a di-Tyr molecular “staple”, could be more stable than pure non-covalently stabilized trimer solely due to entropic reasons.

**Figure 8:**
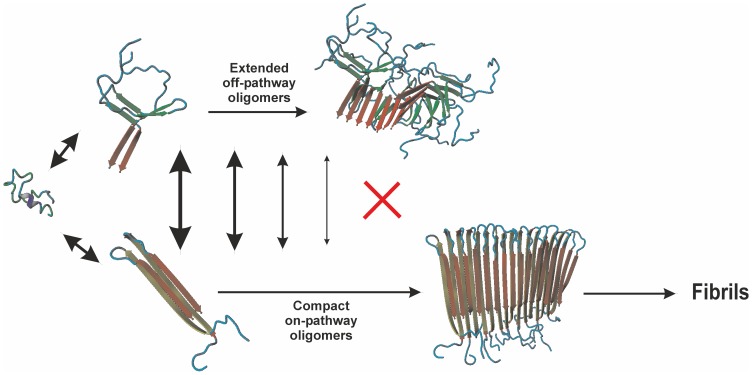
Oligomer evolution scheme. Oligomer evolution scheme showing an on-pathway scenario, leading to fibrils, and off-pathway scenario.

In our work the observed effect of a cross-link was a preferential stabilization of compact oligomeric structures as contrasted to extended forms which are also detected in IMS-MS experiments. Although IMS-MS is a relatively new technique, it has already been widely used to study oligomerizing peptides/proteins [44], including Aβ peptide [45], [50]–[52]. Similar equilibria of different structural variants of oligomers of the same order, coexisting in solution, have already been detected for other peptides/proteins [44], [53], [54]. Shifts in equilibrium between different oligomeric forms may influence further evolution of oligomeric forms. In case of Aβ the evolution is believed to follow an on-pathway scenario, leading to fibrils, however accompanied by off-pathway species (**Figure 8**). In consequence, stabilization of compact species would lead to smaller fraction of off-pathway species. If these species, as suggested by some authors, are the main neurotoxic oligomeric forms, the cross-link should then be expected to decrease their number. On the other hand stabilization of fibrils, thought to provide a reservoir for decomposition to oligomers, can decrease their turnover rates and increase their accumulation indirectly leading to the increase of the levels of oligomeric species.

Ion Mobility-Mass Spectrometry allows to characterize molecular shapes in the gas phase. The issue of the correspondence of these results to in-solution structures is a general problem for IMS studies of biological samples, frequently discussed as the field grows fast [55]. In case of proteins it has been shown that ESI can retain solution phase structures at least for some time, as ESI-MS analyses reproduce several known protein structural distributions and stoichiometries [56]. For smaller objects like peptide oligomers the time frame of the re-equilibration into gas phase conditions is not yet known and thus the level of changes in the population of detected species caused by the stripping of water cannot be estimated. However, in a parallel study of metal-bound Aβ1-40 oligomers [Sitkiewicz et al., Journal of Molecular Biology, accepted], we have observed that monomers stripped of metal during ESI indeed do equilibrate to apo-state distribution, while dimers and trimers do not, retaining the metal-bound drift time profile in their metal-free form. This observation indicates that the “structural memory” of Aβ oligomeric structures during ESI can last longer than the time of IMS experiment. Even if forms found in the gas-phase become minor species in solution, it can also be argued that their presence may be of importance in case of AD as the disease develops slowly, indicating dependence on rare species.

As the molecules in the gas phase become stripped of water the hydrophobic effect is no longer present and in parallel the electric forces become strengthened due to decreased dielectric constant of the environment. Moreover, in positive ion mode the neutral pH, in-solution charge of Aβ1-40 (−3) becomes substituted by a positive charge. We have noted however that the measurements in negative mode, which could better reproduce the in-solution charge distribution do not change the drift time profiles while leading to poor sensitivity [Sitkiewicz et al., Journal of Molecular Biology, accepted]. Also, the analyses of α-synuclein have shown that the ionization mode polarity has no effect on the distribution of monomeric conformers of this protein, nor on the distribution of monomer and dimer species formed [57]. As long as the charge is relatively low its polarity seems not to influence the collisional cross-section measured, whereas at higher charges of any polarity the electrostatic repulsion readily generates more expanded forms in the gas phase, as was also observed in the presented work. This advocates for focus on low-charge structures in IMS-based studies of biological molecules.

Characteristic patterns of well resolved IMS-MS signals split in the domain of drift time were observed before for basic *in vitro* preparations of Aβ peptide and its different variants, using both negative and positive ionization. The signals were assigned to specific oligomeric species following the analysis of their corresponding isotopic envelope spacing ([45] and the present work) or without such analysis [51], [52], [58], although unequivocal identification of species requires the analysis of well resolved isotopic envelopes. For this reason in some cases, contradictory assignments were obtained as discussed in detail in ref. [45]. Drift time measurement allowed to resolve species with the same or very similar m/z value, which could not be distinguished in the classic MS spectrum. In the case of Aβ peptide, some oligomers of different order have an overlapping isotopic envelope (for instance, the m/z values of MON^2+^, DIM^4+^, TRI^6+^ and TET^8+^ are the same) and can be only seen as a separate species after additional ion mobility separation. Similarly, different structural forms of the same oligomer will have the same m/z value, but might be discernible by their collisional cross section. To determine unambiguously whether a particular signal originates from a structural variant of an oligomer of the same order or oligomer of different order which happens to have the same m/z value, a well-resolved isotopic envelope is essential. In the present work, all identification was based on isotopic envelope analysis. It allowed us to prove the coexistence of compact-extended structural forms of a dimer, signals from which were previously improperly assigned to dimer/tetramer pairs, as in the case of DIM^5+^ (Figures 1c, 2d in [50]). In the cited work, negative ionization was used, whereas we used positive ionization. However, we have replicated measurements of Aβ peptide in the negative ionization mode [Sitkiewicz et al., Journal of Molecular Biology, accepted]. The analysis of isotopic envelopes present in the resulting negative ionization spectra fully confirms the assignments presented in this paper and shows that the results of IMS-MS analysis are the same regardless of on the ionization mode.

Di-Tyr linked Aβ oligomers were previously obtained by Cu- or HRP-mediated reaction [41], [46]. In the latter work, 14 µg/ml Aβ in reaction with 40 µg/ml HRP, 3 µM H_2_O_2_ yielded only higher order oligomers and poorly resolved lower-order oligomeric species. However, our studies, conducted under comparable conditions (30 µM concentration of Aβ, 45 µg/ml HRP at 20 µM H_2_O_2_), showed very well separated lower-order oligomeric species. In [39], where fibrillar starting material was used, only higher order oligomers were present after reaction. When a monomeric starting material was used, a spectrum of low order oligomers were observed, poorly resolved by PAGE. Metal-induced di-Tyr formation protocols also led to inseparable higher order oligomeric species (see Figure 4 in [27]), however more recent protocols [37] show results similar to obtained in our study. The reason for this differences is not clear; our results confirm that using HRP/H_2_O_2_ affords a well-defined population of low order oligomers that form with high efficiency.

In conclusion the compact-extended assignment is based in this work on unequivocal measurement of the masses of underlying species, and an alternative identification of species must be ruled out. IMS-MS proved its unique value for structural studies of oligomerizing systems, as it allows the separation of different structural variants co-existing in solution even in complex mixtures. In the present work, the level of complexity was elevated by introduction of a covalent cross-link which significantly increased the number of possible forms. We nevertheless managed to obtain the structural information, in the form of collisional cross-section values, for each of the forms that could be detected. The possibility to gain such insight into the structural properties opens the way for verification of structural models of Aβ peptide oligomers, some of which are expected to be major synapto/neurotoxic agents in AD.

## Materials and Methods

### Materials

The Aβ peptide was obtained by expression in *E. coli* and purified by HPLC as described previously [59]. The identity of Aβ1–40 was verified using a Q-ToF Premier ESI-MS instrument (Waters Corp., Milford, MA). Typically, the concentration of the peptide in the stock solution was within the range of 30–80 µM and, when necessary, pH was adjusted with ammonia. The stocks were stored at 4°C and used within 48 h. Horseradish peroxidase (HRP) was purchased from Sigma; H_2_O_2_ from Riedel-de Haën; NaOH, HCl, Tris were obtained from Merck and ammonium acetate from Fluka.

### Di-Tyrosine Crosslinking of Aβ Peptides

Di-Tyr cross-links were generated by incubating 30 µM Aβ1-40 with HRP (45 µg/ml) and hydrogen peroxide (20 µM) in 10 mM ammonium acetate, pH 7.4. A stock solution of Aβ1-40 was diluted to the final concentration of 30 µM in the 10 mM ammonium acetate, pH 7.4. All reagents were freshly prepared as described above or stored no longer than two days. Concentrations of HRP and Aβ1-40 solutions were determined spectrophotometrically (Cary 50 Bio, Varian, Palo Alto) at 280 nm, using molar extinction coefficients of 39,800 M^−1^cm^−1^ for HRP and 1410 M^−1^cm^−1^ for Aβ1-40; the latter value was calculated from the amino acid composition [60].

### Fluorescence Spectroscopy

The reaction product was detected fluorometrically using a fluorescence spectrometer (Cary Eclipse, Varian, Palo Alto) at an excitation wavelength of 315 nm, with a characteristic emission band for di-tyrosine centered at 400–404 nm [47]. Spectra were recorded in 10 mm cuvettes at 350 to 550 nm, 1 nm/s, 25°C. In a control experiment, Aβ1-40 was incubated with HRP or with H_2_O_2,_ or without either of these two reagents.

### SDS-PAGE Gel Analysis

Di-Tyr cross-linked Aβ peptide was analyzed by SDS-PAGE to confirm the presence of oligomers. Samples were collected at 9 time points during the reaction: 0 min, 10 min, 20 min, 30 min, 40 min, 50 min, 60 min, 90 min, and 2 h. Aliquots for each reaction were heated to 95°C for 10 min in electrophoresis sample buffer containing 4% SDS and 2.5% β-mercaptoethanol. Molecular size markers (Mark12, Invitrogen Unstained Protein Molecular Weight Marker, Fermentas) and the Aβ samples were separated on 6–16% polyacrylamide gels and were then either stained with Coomassie Brilliant Blue or transferred to 0.25 µm PVDF membranes (Immobilon-P PVDF Membrane, Millipore ) for immunoblotting.

### Western Blotting

Polyacrylamide gels (6–16%) were subjected to electrophoresis and transferred to PVDF membranes. The membranes were blocked with 10% low fat milk/Tris-buffered saline (0.15 M NaCl, 20 mM Tris, pH 7.4) for 1 h at room temperature. Subsequently, blots were incubated overnight at 4°C with an anti-Aβ monoclonal antibody 6E10 targeting Aβ residues 1–17 (1∶10000; Millipore), or with the primary mouse anti-DT monoclonal antibody 1C3 directed against di-Tyr residues (1∶1000; gift from Y.Kato [61]). After four washes in TBS for 15 min, the blots were incubated with a HRP-conjugated anti-mouse IgG (1∶2000; Sigma, USA) for 2 h at room temperature and then washed again. Blots were developed with ECL reagent (GE Healthcare Life Sciences) for 1 min according to the instructions of the manufacturer, and the chemiluminescent signal was captured on Hyperfilm ECL (GE Healthcare Life Sciences).

### Ion Mobility Spectrometry–Mass Spectrometry (IMS-MS)

Experiments were performed with a Synapt G2 HDMS instrument (Waters Corp., Milford, MA). For IMS-MS analysis the mixture of Aβ1-40 peptide at 80 µM concentration in 10 mM CH_3_COONH_4_, pH 7.4 with HRP and H_2_O_2_ was infused directly to the ion source of a mass spectrometer, with a glass Hamilton syringe through a stainless steel capillary. The injection and IMS measurement were performed continuously for 3 h with flow rate 7 µl/min. The mass signals were measured in the range of *m/z* 400–4100 at the rate of 1 scan/s. The instrument was operated in electrospray positive ion mode with a capillary voltage of 2.8 kV and sample cone voltage of 38 V. The mobility T-wave cell was operated at a pressure of 3.8 mbar of nitrogen, with a wave velocity of 650 m/s and an amplitude of 39 V. Data acquisition and processing were carried out with MassLynx (V4.1) and DriftScope (V2.1) software supplied with the instrument.

The T-wave system allows to define and characterize the relationship between the measured drift time and collisional cross section. Consequently, following the approach taken in other studies, the T-wave mobility drift times were converted to collisional cross-section Ω values for all detected oligomeric forms using a set of proteins of known Ω (myoglobin, cytochrome c, ubiquitin) as callibrating standards as described in previous publication [45].

### Modeling of Covalent Aβ1–40 Oligomers

All the simulations were performed using the Yasara Structure package. The original Yasara 2 forcefield [62] was extended for di-Tyr molecules on the basis of *ab initio* calculations performed with the aid of Firefly (PC GAMESS) version 7.1 [63]. Di-Tyr molecules were at first modeled as the 2,2-, 2,3-, or 3,3- bis-phenol bond, which correspond to the δ-δ, δ-ε or ε-ε cross-links in di-tyrosine, respectively. All these structures were preoptimized using the semi-empirical PM3 method and then analyzed using the DFT B3LYP/6-31G(d,p) method. Corrections for solute-solvent interactions were also introduced by the polarizable continuum model (PCM) [64]. The *QM*-derived bond lengths and ESP charges were used to extend the Yasara 2 forcefield.

Initial protofibril-like structures of a non-covalently stabilized hexadecamer of Aβ1–40 was prepared with the aid of combination of constrained simulated annealing and molecular dynamics, as described previously [45]. The following constraints deduced from accessible fibril structural information was used: intermolecular D23–K28 salt bridge; residues L17–V24 and A30–V39 kept in the extended conformation to build a β-sheet core; the twist of the backbone in the turn region; the intrachain distance constraints (2.9–8.5 Å between Cα atoms) set in agreement with mutational data for F19–G38 and A21–V36 residue pairs to simulate their spatial proximity. For the purpose of the present work, a central hexamer (peptide molecules 6–11) was extracted, modified according to the assumed di-tyr cross-linking type, placed in a water box and subjected for 50 ns to molecular dynamics (MD) in the NTP ensemble (T = 298 K and p = 1 atm). The weights of all constraints were iteratively decreased; the last 20 ps MD was performed without any constraints.

## Supporting Information

Figure S1 **Control SDS PAGE gels**. SDS PAGE obtained at different times of incubation of Aβ with H_2_O_2_ (A) and horseradish peroxidase (B) visualized by Western Blotting with anti-Aβ monoclonal antibody 6E10.
(TIF)

Figure S2 **Results of fitting of a set of Gaussian curves**. Results of fitting of a set of Gaussian curves to simulate the drift time profiles obtained before (red) and after (blue) crosslinking. Figures represent the quality of the fit and Table show numeric parameters of the fit. The same set of curves, characterized by the same drift times, were used to fit to profiles before and after crosslinking; only their amplitudes changed.
(TIF)
