# Supplementary figures and images for: Di-Tyrosine Cross-Link Decreases the Collisional Cross-Section of Aβ Peptide Dimers and Trimers in the Gas Phase: An Ion Mobility Study
Source: PLoS One. 2014 Jun 19;9(6):e100200. doi: 10.1371/journal.pone.0100200 (PMC4063900; doi:10.1371/journal.pone.0100200)

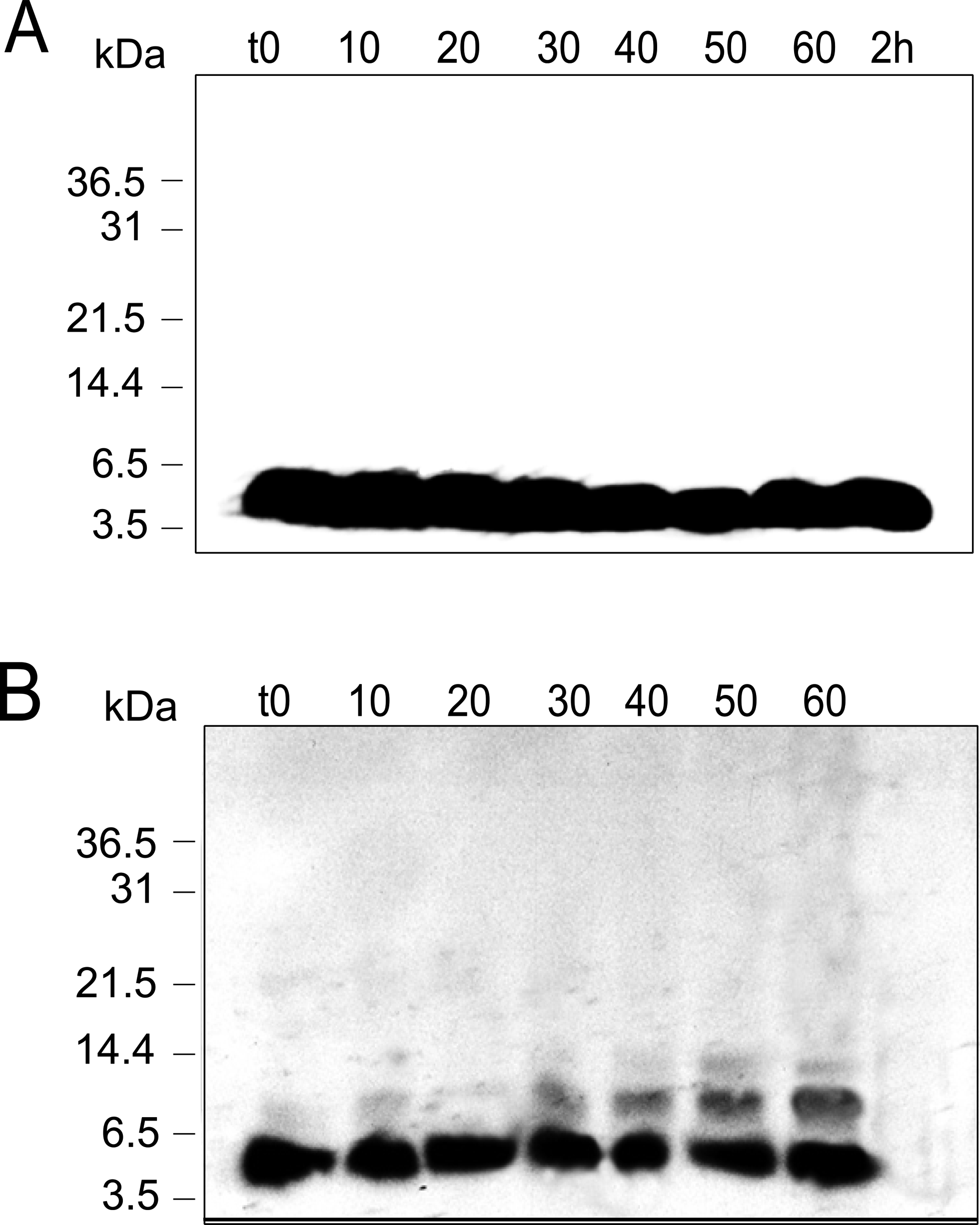

Supplement: Figure S1 — Control SDS PAGE gels. SDS PAGE obtained at different times of incubation of Aβ with H2O2 (A) and horseradish peroxidase (B) visualized by Western Blotting with anti-Aβ monoclonal antibody 6E10. (TIF) [file pone.0100200.s001.tif]

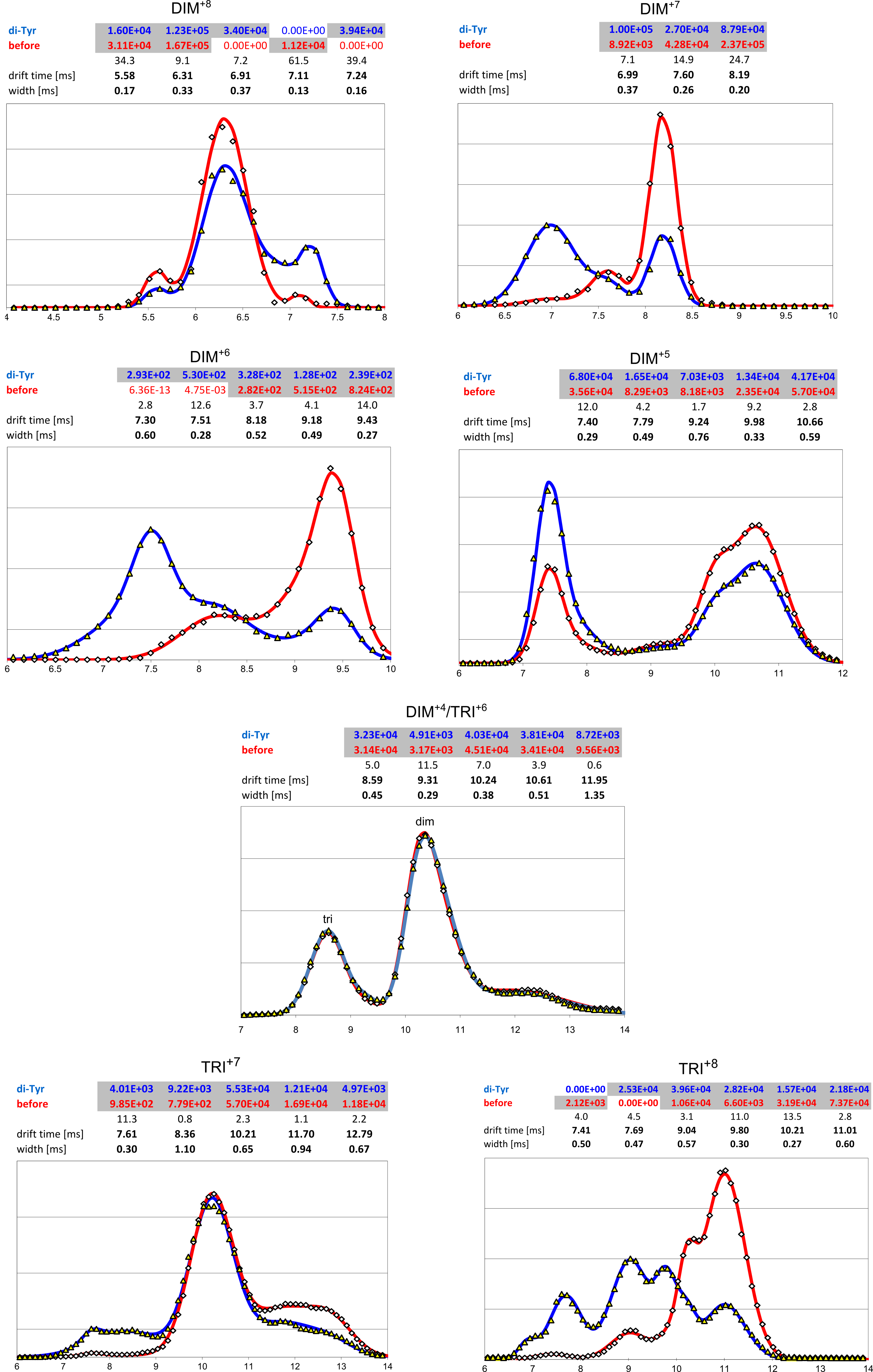

Supplement: Figure S2 — Results of fitting of a set of Gaussian curves. Results of fitting of a set of Gaussian curves to simulate the drift time profiles obtained before (red) and after (blue) crosslinking. Figures represent the quality of the fit and Table show numeric parameters of the fit. The same set of curves, characterized by the same drift times, were used to fit to profiles before and after crosslinking; only their amplitudes changed. (TIF) [file pone.0100200.s002.tif]
